# Integrative and Complementary Therapies: Do You Know What Your Patients Are Taking?

**DOI:** 10.6004/jadpro.2012.3.6.1

**Published:** 2012-11-01

**Authors:** Pamela Hallquist Viale

As we enter the fall season, *JADPRO* is ready to begin publishing our next review series. Our first year highlighted the use of biomarkers in various types of cancer, and our second year provided a comprehensive look at the toxicities and adverse events advanced practitioners (APs) encounter in practice. Our third series topic involves integrative therapies and complementary and alternative medicine (CAM). Many patients in our practices have used CAM or are considering the use of integrative therapies. In fact, the 2007 National Health Interview Survey (NHIS) demonstrated that approximately 38% of adults use CAM at one time or another. With this series, we at *JADPRO* hope to increase the AP’s understanding of integrative therapies and their place (or lack thereof) in the care of the patient with cancer.

## Defining Integrative and CAM Therapies

It is somewhat difficult to describe CAM therapies accurately. The National Center for Complementary and Alternative Medicine (NCCAM) at the National Institutes of Health (NIH) defines CAM as "a group of diverse medical and health-care systems, practices, and products that are not generally considered part of conventional medicine" (NCCAM, 2012, p. 1). The Arizona Center for Integrative Medicine (2012) defines the practice as healing-oriented medicine that incorporates the whole person, including lifestyle, therapeutic relationship between provider and patient, and evidence. The principles of integrative medicine state that appropriate use of conventional and alternative methods can affect healing responses, and that natural and less invasive approaches are preferred (Arizona Center for Integrative Medicine, 2012). Therapies vary widely and can include dietary supplements, massage, or even mind-body therapies.

## PC-SPES: A Cautionary Tale

In my own practice, the use of dietary supplements came up frequently. It was important to remain nonjudgmental regarding supplements. First, I wanted to know exactly what my patients were taking so I could determine any potential for drug-substance interactions; I believe the patient-provider relationship was strengthened by this knowledge exchange. However, in one case, the use of dietary supplements in a single patient revealed the possible negative effects that specific therapies could have.

In late 2001, my 64-year-old patient with prostate cancer progressed after androgen deprivation therapy and chemotherapy; he became notably depressed. At a subsequent visit, his spirits were higher, and he reported feeling better and less anxious over his disease; his prostate-specific antigen (PSA) test showed lower values. He’d started taking a supplement without telling the treatment team, yet his positive reaction to the therapy prompted him to share his finding with us. The patient was taking PC-SPES, a supplement manufactured by BotanicLab in Brea, California, containing a combination of eight herbs, including chrysanthemum, isatis, licorice, Panax pseudo-ginseng, and saw palmetto, several of which had shown antitumor activity (Kosty, 2004; Sovak et al., 2002). This supplement was marketed as a "prostate health" product, and recommendations for therapy included taking three to six capsules a day on an empty stomach; a bottle of 60 capsules cost $108 (Kosty, 2004).

The substance was evaluated in several clinical trials, showing efficacy in the reduction of PSA levels and the shrinkage of some tumors. However, a subsequent review of PC-SPES showed contamination of product, with some lots containing indomethacin and diethylstilbestrol (DES); warfarin was also detected in specific lots, amounting to about 1.5 mg/day in nine capsules (Sovak et al., 2002). A clinical trial examining PC-SPES in prostate cancer was halted after the synthetic estrogen was found. An additional contaminant, alprazolam, was discovered in several lots of a companion product called SPES, with equivalent amounts of approximately 1 mg of alprazolam a day (Kosty, 2004; Strax, 2002).

Obviously, these contaminants had the potential to create harm for the patient; drug-drug interactions and increased toxicity could have resulted, seriously impacting patient health. The contamination led to the withdrawal of the product from the market, despite the previous efficacy of the product shown in clinical trials. A phase II trial led by Small et al. with initial results presented at the 2002 American Society of Clinical Oncology (ASCO) annual meeting demonstrated a promising 47% response rate with PC-SPES compared with DES (Small et al., 2002). However, the study was stopped because of the contamination of drug(s) in the product (Reynolds, 2002).

The problems seen with PC-SPES underscore the importance of research and the challenges encountered in the study of natural compounds and supplements. However, this research is sorely needed if we are to elevate these products to the realm of accepted medical treatment.

## The Need for Education

My hope is that our upcoming series will not only educate the AP about possible therapies used in integrative medicine and CAM, but also highlight available research findings as well. Integrative medicine and CAM therapies are here and being used by our patients. Therefore, increasing our knowledge about unconventional medicine and therapies is necessary. And although the dietary supplement regulations are not as rigorous as the regulations for prescription or over-the-counter drugs, research is needed to determine the effectiveness of integrative medicine and CAM therapies in patients with cancer.
